# The molecular characters and antibiotic resistance of *Clostridioides difficile* from economic animals in China

**DOI:** 10.1186/s12866-020-01757-z

**Published:** 2020-03-30

**Authors:** Wen-Zhu Zhang, Wen-Ge Li, Yu-Qing Liu, Wen-Peng Gu, Qing Zhang, Hu Li, Zheng-Jie Liu, Xin Zhang, Yuan Wu, Jin-Xing Lu

**Affiliations:** 1 State Key Laboratory of Infectious Disease Prevention and Control, National Institute for Communicable Disease Control and Prevention, Chinese Center for Disease Prevention and Control, Beijing, China; 2 Institute of Animal Science and Veterinary Medicine, Shandong academy of agricultural Sciences, Jinan, China; 3 Department of Acute Infectious Diseases Control and Prevention, Yunnan Provincial Centre for Disease Control and Prevention, Kunming, China; 4 Regional Center for Disease Prevention and Control, Aksu, Xinjiang China; 5 Collaborative Innovation Center for Diagnosis and Treatment of Infectious Diseases, Hangzhou, China

**Keywords:** *Clostridioides difficile*, Economic animal, Antibiotics resistance

## Abstract

**Background:**

It has been performed worldwidely to explore the potential of animals that might be a reservoir for community associated human infections of *Clostridioides difficile*. Several genetically undistinguished PCR ribotypes of *C. difficile* from animals and human have been reported, illustrating potential transmission of *C. difficile* between them. Pig and calf were considered as the main origins of *C. difficile* with predominant RT078 and RT033, respectively. As more investigations involved, great diversity of molecular types from pig and calf were reported in Europe, North American and Australia. However, there were quite limited research on *C. difficile* isolates from meat animals in China, leading to non-comprehensive understanding of molecular epidemiology of *C. difficile* in China.

**Results:**

A total of 55 *C. difficile* were isolated from 953 animal stool samples, within which 51 strains were from newborn dairy calf less than 7 days in Shandong Province. These isolates were divided into 3 STs and 6 RTs, of which ST11/RT126 was predominant type, and responsible for majority antibiotic resistance isolates. All the isolates were resistant to at least one tested antibiotics, however, only two multidrug resistant (MDR) isolates were identified. Furthermore, erythromycin (ERY) and clindamycin (CLI) were the two main resistant antibiotics. None of the isolates were resistant to vancomycin (VAN), metronidazole (MTZ), tetracycline (TET), and rifampin (RIF).

**Conclusions:**

In this study, we analyzed the prevalence, molecular characters and antibiotic resistance of *C. difficile* from calf, sheep, chicken, and pig in China. Some unique features were found here: first, RT126 not RT078 were the dominant type from baby calf, and none isolates were got from pig; second, on the whole, isolates from animals display relative lower resistant rate to these 11 tested antibiotics, compared with isolates from human in China in our previous report. Our study helps to deep understanding the situation of *C. difficile* from economic animals in China, and to further study the potential transmission of *C. difficile* between meat animals and human.

## Background

*Clostridioides difficile* is a spore-forming, Gram-positive, anaerobic bacillus found ubiquitously in the environment and the gastrointestinal tracts of humans and animals [1, 2]. *C. difficile* has emerged as the most common infectious pathogen of antibiotic-associated diarrhea (AAD), causing heavy disease economic burden [3]. Many studies have been performed to explore the potential of animals that might be a reservoir for community associated human infections of *C. difficile* around the world [4–6]. Pigs and calves were the most common meat animals for the isolation of *C. difficile*, in which PCR ribotype 078 was recognized to be frequently isolated from pigs and RT033 from cattles, however, there was extremely variations among countries [7–9]. Besides, there are other genetically indistinguishable strains which have been identified in human and animal isolates, such as, RT237, though to only be isolated from pigs in Australia, were reported to be found from *C. difficile* infection (CDI) patients [10]. This finding further supports the potential transmission of *C. difficile* from animals to human. In addition, high intestinal colonization percentages of up to 25% have been found in families and employees living and working on pig farms [11].

In China, until now, only one study focusing on *C. difficile* from animals has been published, however, in which calf was not included [12]. Here, we studied the prevalence, molecular characters and antibiotic resistance of *C. difficile* from calf, sheep, chicken, and pig. This study helps to deep understanding the situation of *C. difficile* from economic animals in China, and to further study the potential transmission of *C. difficile* between meat animals and human. In future, further studies on the genetic relationship between animals and human *C. difficile* strains are required to help better understanding its role in transmission of this pathogen.

## Results

### *C. difficile* isolates from economic animals in China

A total of 55 *C. difficile* strains was isolated from 953 fresh stool samples of economic animals, including sheep, cow, pig, and chicken, at a rate of 5.77%. Details were summarized in Table 1. Most of the *C. difficile* isolates were isolated from the feces of calves (aged < 7 days of age) with rate of 43.22% (51/118) in Shandong province (Table 1). Within the 51 isolates, 5 strains are from two different batch of N and ND with a rate of 4.24%, while the rest 46 isolates are from SN and YCVTN with a rate of 38.98% (Table 1). None *C. difficile* isolates was isolated from adult economic animals, except for 4 *C. difficile* isolates were obtained from 200 feces samples of adult sheep (Table 1).

**Table 1:** Isolation of *C. difficile* from China economic animals

Animal	Region	No. of specimens	Age group	No. of *C. difficile* isolates
Sheep	Yunnan	200	Adult	4
Cow	Yunnan	200	Adult	0
	Shandong	118	Calf (< 7 days)	51
	Shandong	57	Calf (7 days~ 1 mouth)	0
Pig	Yunnan	200	Adult	0
	Shandong	120	Piglet (23 days~ 65 days)	0
Chicken	Shandong	58	–	0
Total		953		55

### Molecular characters of *C. difficile* isolates from economic animals in China

All the 55 *C. difficile* isolates were positive for genes *tcdA* and *tcdB* (A+ B+), among which 92.73% (51/55) were also positive for the binary toxin genes (CDT+). And the rest 3 isolates from Yunnan adult sheep and 1 isolate from young calf in Shandong were CDT- (Table 2). Most of the isolates (50/51) from calve below 7-days-old were CDT+ (Table 2).

**Table 2:** The molecular features and antibiotic susceptibility of 55 *C. difficile* isolates

Source	NO.	STs	RTs	Toxin				No. of resistant isolates / Clinical breakpoints										
				***tcdA***	***tcdB***	***cdtA***	***cdtB***	MXF	CLI	TET	ERY	LVX	CIP	CHL	MEM	VAN	MTZ	RIF
								≥8	≥8	≥16	≥8	≥8	≥8	≥32	≥16	≥4	≥32	≥4
SN	1	11	ICDC028 (RT078)	+	+	+	+	0	0	0	1	0	1	0	0	0	0	0
SN	21	11	ICDC035 (RT126)	+	+	+	+	0	14	0	21	0	2	0	0	0	0	0
SN	7	11	ICDC050	+	+	+	+	0	4	0	7	0	1	0	0	0	0	0
SN	6	11	ICDC052	+	+	+	+	0	1	0	6	0	2	0	0	0	0	0
N	4	11	ICDC035 (RT126)	+	+	+	+	0	3	0	4	0	2	0	0	0	0	0
YCVTN	1	11	ICDC028 (RT078)	+	+	+	+	0	0	0	1	0	1	0	1	0	0	0
YCVTN	9	11	ICDC035 (RT126)	+	+	+	+	0	6	0	9	0	3	0	0	0	0	0
YCVTN	1	11	ICDC050	+	+	+	+	0	0	0	1	0	0	0	0	0	0	0
YNY	1	11	ICDC035 (RT126)	+	+	+	+	0	1	0	0	0	0	0	0	0	0	0
ND	1	3	ICDC039 (RT220)	+	+	–	–	0	1	0	1	0	1	1	0	0	0	0
YNY	3	468	ICDC094	+	+	–	–	0	1	0	0	0	2	0	0	0	0	0
Total	55							0	31	0	51	0	15	1	1	0	0	0

**Fig. 1:**
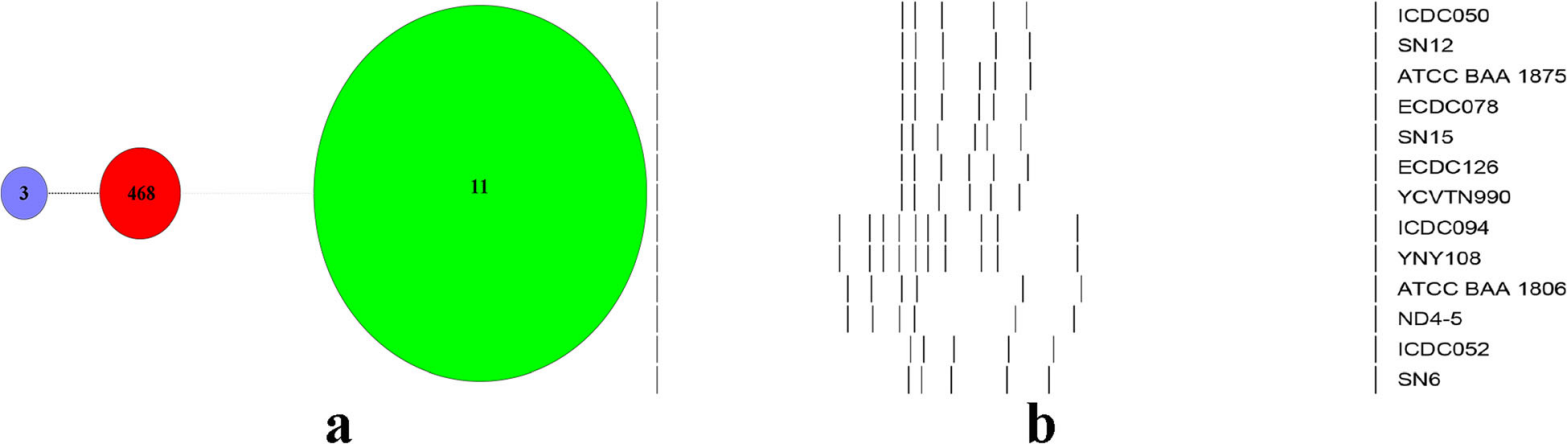
The minimum spanning tree and PCR ribotypes (RTs) pattern of *C. difficile*. **a** Relationship of the 55 colonizing *C. difficile* strains by minimum spanning tree based on MLST data. Each circle corresponds to ST types, the number of which is indicated for the size of circles. The green color circle belongs to the clade5, the red and purple color circles belong to the clade1. The dotted lines between circles indicate the similarity between profiles (black, 3 alleles; gray, 7 alleles). **b** PCR ribotypes (RTs) banding patterns for *C. difficile* ICDC050, RT078 (ECDC078, ATCC BAA 1875), RT126 (ECDC126), ICDC094, RT220 (ATCC BAA 1806) and ICDC052 when visualised by BioNumerics version 7.6

According to the multilocus sequence typing (MLST) [13], the total 55 *C. difficile* isolates belong to 3 MLST genotypes, in which sequence type (ST) 11 (51/55) was the most predominant one (92.73%), followed by ST468 and ST3 (Table 2). The new ST468 identified in our study, was only present in samples from sheep in Yunnan. All the *C. difficile* isolates in this study were divided into 2 clades as follows: clade1 (*n* = 4) and clade5 (*n* = 51) **(**Fig.1a).

In addition, all the 55 *C. difficile* isolates were divided into 6 different PCR ribotypes (RTs) according to the capillary electrophoresis based on QIAxcel [14] as follows: ICDC028 (*n* = 2), ICDC035 (*n* = 35), ICDC039 (*n* = 1), ICDC050 (*n* = 8), ICDC052 (*n* = 6) and ICDC094 (n = 3) **(**Fig.1b and Table 2). To be clear that ICDC028 in our study was the same as RT078, and ICDC035 was identical with RT126 **(**Fig.1b and Table 2).

### **A**ntibiotics resistance profile of these *C. difficile* strains

**Fig. 2:**
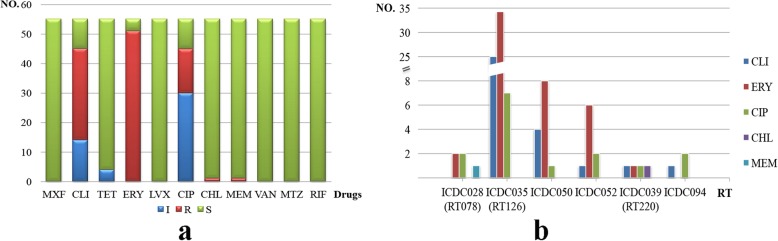
The resistance characteristics and the relationship with RTs of the 55 *C. difficile* strains. **a** The resistance characteristics of all 55 *C. difficile* strains. The name of 11 tested drugs in the horizontal axis. For the vertical line, the numbers refer to number of strains involved in S (susceptible), I (intermediate) and R (resistant). **b** Different resistant number to 5 tested drugs among each RT group. CLI, ERY, CIP, CHL and MEM were included because at least one RT was resistant to them

All the 55 *C. difficile* isolates were tested for their minimal inhibitory concentration (MIC) against 11 antimicrobial agents. As a result, they were all resistant to at least one antibiotics tested (Table 2 and Fig. 2a). Moreover, they all displayed higher resistant rate to macrolide-lincosamide-streptogramin B (MLSB), CLI and ERY, with a resistance rate at 53.36 and 92.73%, respectively (Table 2 and Fig. 2a). It is known that most isolates from human showing resistance to fluoroquinolones (FQs) in our previous study. Interestingly, all the isolates in this study are susceptible to moxifloxacin (MXF) and levofloxacin (LVX), except 15 isolates are resistant to ciprofloxacin (CIP) (Table 2 and Fig. 2a). There was only one each isolate resistant to chloramphenicol (CHL) and meropenem (MEM), respectivley (Table 2). None of the isolates were resistant to VAN, MTZ, TET, and RIF in this study (Table 2 and Fig. 2a). On the whole, isolates from animals display relative lower resistant rate to these 11 tested antibiotics, compared with isolates from human in China in our previous report [15].

It is indicated that RT126/ICDC035 was the main type in our study, and all the isolates with this type were resistant to at least one antibiotics (Table 2 and Fig. 2b). In addition, two isolates with RT078/ICDC028 were all resistant to both ERY and CIP, and one of them was also resistant to MEM (Table 2 and Fig. 2b). There were two isolates, ST3/RT220 and ST11/RT078, were confirmed as MDR, according to the definition. Furthermore, the MDR profile were CLI/CIP/ERY/CHL and ERY/CIP/MEM, respectively (Table 2 and Fig. 2b).

## Discussion

Considering the potential zoonotic transmission of *C. difficile* from meat animal to humans, many studies have been performed to study the carriage of *C. difficile* in both economic and companion animals [11, 16–18]. Studies in North America reported the presence of *C. difficile* in food animals and meat with rates up to 42% [19]. Importantly, pig and calf were thought to be the predominant animals carrying *C. difficile* isolates*.* However, such kind of study is quite limited in China, although there are many molecular studies of clinical *C. difficile* in China, even in the Asia-Pacific area [20, 21]. Until now, there is only one report on *C. difficile* from pig, chicken and duck in China in 2019, but calf was not included [12]. Therefore, in this study, we determined to analyze the prevalence and genotypic characters of *C. difficile* from economic animals in China, including dairy calf, pig, sheep and chicken, which help to fully understand the situation of *C. difficile* from animals in China.

It is known that age is a key important factor affecting the isolation rate of *C. difficile* from animals, with a much higher prevalence in newborn than in adult animals [22]. Similarly, most of the *C. difficile* isolates (51/55) identified in this study, were from newborn dairy calf within 7 days. While this age effect in pigs and calves has been repeatedly reported in several studies [17, 23, 24], the reasons and the main sources for this high colonization in the first stage of life remain unknown. In a previously study from Belgium, the prevalence rate of *C. difficile* isolates from calves and adult cattle is 11.3 and 5.5%, respectively [25]. Although there was a higher probability of colonization in calves of less than 6 months in age than in cattle over 11 months of age [25], but none *C. difficile* isolates were identified from calves with age between 7 days to 1 month in our study. Furthermore, there were only 4 isolates from adult sheep, which is a little bit lower than that in Australia [5]. Most importantly, no *C. difficile* isolates were obtained from pig (which were thought to be the reservior of *C. difficile*), and chicken in our study. However, the carriage rate of that in Europe, North American and Australia is around 29.6% ~ 67.2% [26, 27]. Why no isolates are obtained from pig feces samples? It might be attributed to that the quality of feces samples collected, and the situation /location of the pig farm. In our next study, we may include more pig farms with distinct locations, and use the anaerobic swab or broth to carry the samples on site.

It is generally accepted that some PCR ribotypes seem to be more often associated with a particular animal host, such as RT078 in pigs [7–9], and RT033 in calves [28–30]. However, great diversity has been documented according to geographic locations. In Netherlands, RT012 was most prevalent in cattle in 2012, as expected, in pig samples RT078 predominated (77.8%), being the most reported type in pigs worldwide [19]. In observations from the USA and Canada, RT078 was also reported from calf samples [7, 31]. In addition, 4 RTs (127, 288, 033, and 126) with binary toxin-positive accounts for 70.3% (71/101) of isolates from a calf farm in Australia [32]. In this study, three STs (ST11, 3, and 468) and 6 RTs (RT126, ICDC050, ICDC052, ICDC094, RT078, and RT220) were identified. ST11 (51/55, 92.7%) and RT126 (35/55, 63.6%) were predominant molecular types. Besides, a new type, ST468 was found in our study. Different from our results, prevalence of RT126 from calf in Australia is about 2–6% [16, 32], and in a recent study from Germany is 4% [9]. Zidaric et al. [30] found that RT126 and RT078 predominated in calf on a single veal farm in Belgium. Interestingly, RT126 has also been found in 20% of pigs in Germany [26]. Prevalence of RT126 in humans is relatively low, accounting for 3% of infections in a 2008 European survey [33], however, RT126 is one of the most frequently isolated in humans in Spain [8]. There also are reports of increasing incidence in Taiwan [34], as well as small numbers recovered from patients in Kuwait [35] and Australia [36]. RT126 shares a very similar banding pattern with RT078, and these two RTs are often grouped together. Indeed, it is normally considered as a variant of 078 and has been reported in river water and different animal species in many countries [37, 38], indicating the possible zoonotic potential of this ribotype [6, 26]. In our study, there were 51 out of 55 isolates are *tcdA*, *B* gene and binary toxin gene positive, which are consistent with previous reports around the world that majority of the animal *C. difficile* isolates are toxigenic [8, 18].

The antibiotic susceptibility test displayed that none isolates were resistant to MXF, LVX, TET, RIF, VAN, and MTZ. While these isolates showed high resistant rate to ERY (92.73%), followed by CLI (53.36) and CIP (27.2%). The *C. difficile* isolates from economic animals in our study showed relative high resistance rate to ERY than other countries (45.5–52.9%) [12, 38]. In addition, the majority isolates resistant to ERY and CLI are RT126. The resistant rate to TET varies a lot among different studies. For example, *C. difficile* isolates from pig resistant to TET reached as high as 77.3% in a previous study in China, and similar 76.5% resistance rate in a Spain study [38]. Until now, almost none *C. difficile* isolates from meat animals are resistant to MTZ and VAN, including our study [15], which is a little bit different from drug resistant profile of human that susceptibility to MTZ is decreasing, and even resistant isolates were found [39]. Although all *C. difficile* isolates were resistant to at least one tested drug in our study, but only two MDR isolates were found, indicating lower MDR rate than that in human [40]. Furthermore, *C. difficile* isolates are resistant to less type of drugs compared with our previous study from human [15].

## Conclusions

We explore the prevalence, molecular features, and antibiotic resistance of *C. difficile* from economic animals in China. This study helps to deep understanding the situation of *C. difficile* from economic animals in China, and to further study the potential transmission of *C. difficile* between meat animals and human.

## Methods

### Sample collection

During 2017 and 2018, we collected 953 fresh stool samples from economic animals from Shandong and Yunnan provinces, which including 200 sheep samples, 375 cow samples, 320 pig samples, and 58 chicken samples (Table 1). All animal’s stool samples were authorized and obtained from the farm and the relevant units.

In August 2017, we collected 200 samples from adult sheep (YNY), cows (YNN), pigs (YNZ) in Yunnan, all stool samples were cryopreserved after collection and delivered rapidly to the laboratory. The other stool samples were collected in Shandong province. In October 2017, we collected 79 from dairy calves (N, ND), 120 from pigs (PD), and 58 from chickens (CL) in Jinan. In addition, 50 stool samples (SN) and 46 stool samples (YCVTN) from dairy calves were randomly collected from the farm in Yucheng in 2017–12 and 2018–5. All fecal specimens were added directly to an enrichment broth (Cooked Meat Medium, Oxoid, UK) containing gentamicin (5 mg/L), cycloserine (250 mg/L) and cefoxitin (8 mg/L). Put them in the anaerobic bag (mitsubishi gas chemical company INC., GENBAG, BioMerieux, France), brought to the laboratory, and incubated in an anaerobic jar (Mart, NL) at 37 °Cfor 48 h.

### Isolation of *C. difficile*

1 ml of enrichment broth was alcohol shocked with an equal volume of absolute ethanol for 1 h. A volume of 100 μl supernatant was plated directly on selective cycloserine-cefoxitin-fructose agar plates (CCFA, Oxoid, UK) with 5% egg yolk after ethanol shock treatment and incubated in an anaerobic jar at 37 °C for 48 h. Isolation of *C. difficile* was based on previously described methods, suspected colonies were further confirmed by the 16S rRNA gene [41]. All colonies were cultured on anaerobic medium in an anaerobic environment at 37 °C.

### Multilocus sequence typing (MLST), toxin gene profile, and PCR-Ribotyping

The methods of MLST, toxin genes profile and PCR-Ribotyping was obtained according to previously reported [13, 41]. The primers and the amplification conditions used for PCR ribotyping have been described previously [42].

### Antimicrobial susceptibility test

The minimum inhibitory concentrations (MICs) for 11 antimicrobial agents were determined by Etest strips (bioMérieux, France, and Liofilchem, Italy) on Brucella agar plates (Oxoid, Basingstoke, UK) containing 1 mg/L vitamine K1, 5 mg/L chlorhematin and 5% defibrinated sheep blood, according to the manufacturer’s instructions.

*C. difficile* isolates were tested for susceptibility to MXF, VAN, CLI, TET, ERY, RIF, LVX, CHL, MTZ, CIP and MEM using E-test strips (Biomerieux, France, and Liofilchem, Italy). The breakpoints for antimicrobial agents are shown in Table 2 and are based on recommendations of the European Committee on Antimicrobial Susceptibility Testing (EUCAST) (http://www.eucst.org) and the Clinical and Laboratory Standards Institute (CLSI) M11-A8 and M100-S28, were determined according to a previous study [40, 43, 44]. MDR were defined as resistance to at least three antimicrobial classes. *C. difficile* ATCC 700057 was included as a control in each experiment.

## Data Availability

The data generated and/or analyzed during the current study are available from the corresponding author on reasonable request.

## Abbreviations

PCR: Polymerase chain reaction
RT: Ribotype
MLST: Multilocus sequence typing
MDR: Multidrug resistant
